# Effects of a New Combination of Medical Food on Endothelial Function and Lipid Profile in Dyslipidemic Subjects: A Pilot Randomized Trial

**DOI:** 10.1155/2019/1970878

**Published:** 2019-01-06

**Authors:** Francesco Landi, Anna Maria Martone, Sara Salini, Beatrice Zazzara, Riccardo Calvani, Emanuele Marzetti, Antonio Nesci, Angela Di Giorgio, Bianca Giupponi, Luca Santoro, Angelo Santoliquido

**Affiliations:** ^1^Department of Geriatrics, Neurosciences and Orthopedics, Fondazione Policlinico Gemelli IRCCS, Roma, Italy; ^2^Università Cattolica del Sacro Cuore, Roma, Italy; ^3^Division of Vascular Medicine, Department of Medicine-Fondazione Policlinico Universitario A. Gemelli IRCCS, Roma, Italy; ^4^Medicina d'Urgenza e Pronto Soccorso, Fondazione Policlinico Universitario A. Gemelli IRCCS, Roma, Italy

## Abstract

Nutritional approaches to improve dyslipidemias have been recently developed, but evidences on different medical foods are often incomplete. The main aim of our study was to evaluate the effects on endothelial function, lipid profile, and glucose metabolism of two different combinations of nutraceuticals, first one containing Bergavit (200 mg Citrus bergamia), Omega-3 (400 mg), Crominex 3+ (10 mcg trivalent chromium), and red yeast rice (100 mg; 5 mg monacolin K) and second one containing red yeast rice (200 mg; 3 mg monacolin K), Berberine (500 mg), Astaxanthin (0.5 mg), folic acid (200 mcg), Coenzyme Q10 (2 mg), and Policosanol (10 mg). Fifty subjects affected by dyslipidemia not requiring statin treatment were enrolled in this randomized, blind, controlled trial and submitted to blood sampling for lipid and glucose profiles and instrumental evaluation of endothelial function before and after 6 weeks of treatment with nutraceuticals. Both nutraceutical combinations improved the lipid profile; the nutraceutical containing 5 mg of monacolin K, 200 mg of the extract* Citrus bergamia*, 400 mg of Omega-3, and 10 mcg of trivalent chromium entailed a significant improvement of endothelial function with enhanced cholesterol lowering effect. In conclusion, this study confirms the positive effect of functional food on lipid profile and endothelial function in absence of major undesirable effects.

## 1. Introduction

Cardiovascular (CV) disease is one of the most important public health problems of our time, in both Europe and the rest of the world, accounting for the largest expenditure in the healthcare budgets of many countries [1]. Despite the prevention strategies adopted in the last decades and the growing therapeutic options, CV disease remains the principal cause of disability, morbidity, and mortality [2, 3]. Whereas prevention strategies continue to address individuals who has experienced a major CV event or presented one or more CV risk factors, primordial prevention has been recommended for reaching CV well-being on healthy population [4, 5].

When discussing about major CV risk factors, experts widely recognize important contributions from observational and epidemiological studies, which demonstrate that higher cholesterol levels are associated with greater risks of atherosclerotic CV events [6–8]. In this respect, new nutritional approaches to improve dyslipidemias have been recently developed [9–11]; these products are founded on both amending some potentially dangerous foods and encouraging the intake of specific ‘healthy' medical foods or nutritional supplements. With regard to medical food cholesterol lowering treatment, some evidences suggest that combinations that include more components, recruiting numerous specific mechanisms, are able to better increase the lipid pathway. However, even though these nutraceuticals can be utilized either as substitutions or in addition to drugs [12], the existing evidence on these medical foods is inconclusive.

The present study was, therefore, undertaken to compare the effects of two specific mixtures of nutraceuticals on the control of lipid profile, glucose metabolism and endothelial function among subjects suffering from high levels of serum cholesterol not requiring statin. Our results offer new evidences about the positive effect of functional food on lipid profile and endothelial function.

## 2. Subjects and Methods

We conducted a randomized controlled trial (6-week follow-up) during which participants assumed deferent combination of nutraceuticals (Nutraceutical “A” or Nutraceutical “B”) according to a randomization procedure. All participants gave written informed consent before being enrolled in the study for the prerandomization assessment and at the baseline of intervention for follow-up assessments and treatment.

### 2.1. Study Sample

For the enrollment in the study, we screened individuals admitted to the national campaign, named “Longevity Check-up 7+” (Look-up 7+) and promoted by the Catholic University of Rome [13]. The Catholic University of Sacred Heart Ethical Committee ratified this study protocol within the context of the National Campaigns for CV prevention [14].

The inclusion criteria for the present study were higher level of serum cholesterol not needing statins or statin intolerant, age between 18 and 75 years. The exclusion criteria were the potential side effects to nutraceuticals products, pregnant status, previous prescription of lipid lowering drugs during (6 weeks), and/or severe hypertriglyceridemia (500 mg/dl). Furthermore, we excluded subjects with diseases impairing endothelial function (i.e., uncontrolled diabetes and chronic inflammatory disorders), presence of CV diseases, and subjects treated with vasoactive drugs. Of the total of 75 subjects screened, 17 were excluded for the presence of CV diseases and 3 subjects with uncontrolled diabetes. Five subjects refused to be part of the study protocol. Hence, 67% of the screened subjects (n=50) were fully eligible and entered the study. Finally, three subjects, one in the nutraceutical “A” and two in the nutraceutical “B” group, were missing at the end of the study.

### 2.2. Study Design

Baseline assessments were completed before randomization. After the baseline assessment, participants received nutritional counseling, according to their medical conditions. At the end of this run-in period, participants were randomly stratified according to age and gender by a computer-generated list into two groups assigned to receive Nutraceutical “A” or Nutraceutical “B” for 6 weeks.

Nutraceutical A contained the following: Bergavit (200 mg Citrus bergamia), Omega-3 (400 mg), Crominex 3+ (10 mcg trivalent chromium), and red yeast rice (100 mg; 5 mg monacolin K) (Riscol Plus, Errekappa Euroterapici).

Nutraceutical B contained the following: Policosanol (10 mg), red yeast rice (200 mg; 3 mg monacolin K), Berberine (500 mg), Astaxanthin (0.5 mg), folic acid (200 mcg), and Coenzyme Q10 (2 mg) (Armolipid Plus, RottapharmSpA).

Both nutraceutical “A” and “B” are tablets that were taken by the participants once a day, preferably in the evening. Nutraceutical “A” was chosen as it represents a new combination on the Italian market with an excellent biological plausibility of its main components. At the same time, nutraceutical “B” should be considered the “gold standard”. In fact, this combination has been extensively investigated in several randomized control trials, seven of which were placebo-controlled. Recently, Barrios and colleagues [15], in their review of the clinical evidences, concluded that nutraceutical “B” (Armolipid Plus) has proved to be able to achieve significant reductions in total cholesterol (11-21%) and in LDL-C (15-31%) levels, which is equivalent to expectations from low dose statins.

The primary endpoint was the statistically significant reduction of endothelial dysfunction; secondary endpoints were the modifications in triglycerides, serum total cholesterol (HDL and LDL), blood glucose, and insulin sensitivity index (HOMA index).

### 2.3. Data Collection

All participants enrolled in the study received a dedicated visit comprising an anamnestic questionnaire, the measurement of objective CV health metrics, and the assessment of anthropometric parameters. In particular, the baseline and follow-up assessment were designed to obtain the following data: informed consent, age, gender, lifestyle evaluation (smoking, eating habits, and physical activity), blood pressure measurement, weight and height assessment, waist circumference, lipids measurement and fasting glucose measurements, and plasma level of insulin; furthermore, we estimated the HOMA index using the Matthews' formula [16].

Endothelial function was assessed by evaluating flow-mediated dilation (FMD) of brachial artery that measures the nitric oxide-mediated vasodilation after a period of ischemia (endothelium-dependent vasodilation), in accordance with actual guidelines [17].

All participants were asked to fast and to avoid exercise and vasoactive substances (i.e., drugs, tobacco, and coffee) for at least 12 hours before the examination.

### 2.4. Statistical Analyses

Sample power was previously calculated based on FMD, considering a power of 80% and an *α*-error of 5% with an expected positive result among participants receiving product “A” of 90%, compared to 40% of the subjects in the control group (product “B”). Based on this estimation, 40 subjects were needed to have 80% probability of finding a positive result for the primary outcome from 90% in the subjects taking nutraceutical “A” to 40% in the subjects taking nutraceutical “B”.

Variables were shown as mean values (±SD). Differences in characteristics between control and treatment groups were examined in numerous ways. Quantitative outcomes were analyzed by the Student* t*-test after a previous test for variance homogeneity. The Mann–Whitney analysis was utilized when the normality assumption was not realistic. The Fisher exact test was used for categorical variables. The analysis of covariance (ANCOVA) adjusted for age, gender, and baseline values was used to examine the result of different intervention on lipid pathway and FMD at the end of follow-up period.

All analyses were completed using the SPSS software (SPSS Inc., Chicago, IL, version 11.0).

The funding sources had no part in any phase of study procedures (collection, analysis, and data interpretation).

## 3. Results

Mean age of 47 subjects participating the study was 58.7 (standard deviation 8.7, range from 42 to 75 years) years, and 33 (70%) were men. Characteristics of the study population according to the treatments are summarized in Table 1. At baseline assessment, no differences in life style habits, BMI, and systolic and diastolic blood pressure were observed. Total serum cholesterol, HDL-c and LDL-c, triglycerides, glucose, and insulin were similar between the different groups. None of the participants in the study groups reported difficulty in taking the tablet regarding size, taste, and palatability. Treatment compliance was excellent in both groups and no adverse events were reported.

Mean values of FMD assessment are shown in Table 2. At baseline, FMD were similar between nutraceutical “A” and nutraceutical “B” groups (p=0.14); after the 6 weeks of treatment, FMD in the subjects receiving nutraceutical “A” significantly increases in comparison with subjects receiving nutraceutical “B” (p=0.04). After adjusting for age, gender, and baseline values, FMD value was higher among participants in nutraceutical “A” group compared with subjects in nutraceutical “B” group (p=0.05).

**Figure 1:**
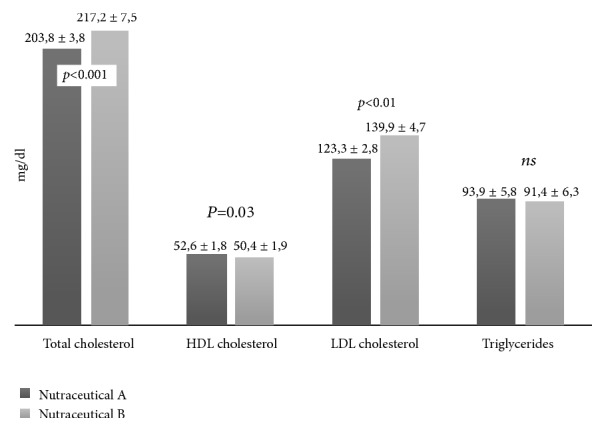
Lipid profile at the end of the study period (ANCOVA analysis adjusted for age, gender, and the baseline values); data are presented as mean ± standard errors values.

Figure 1 shows lipid profile at 6-week follow-up after treatment with nutraceutical “A” and nutraceutical “B”. It is important to highlight that both nutraceuticals produce a noteworthy decrease of serum levels of total and LDL cholesterol, triglycerides, and an improvement of HDL with respect to baseline values (all p<0.01). However, this improvement was more significant in subjects treated with nutraceutical “A” compared with participants receiving nutraceutical “B”.

**Figure 2:**
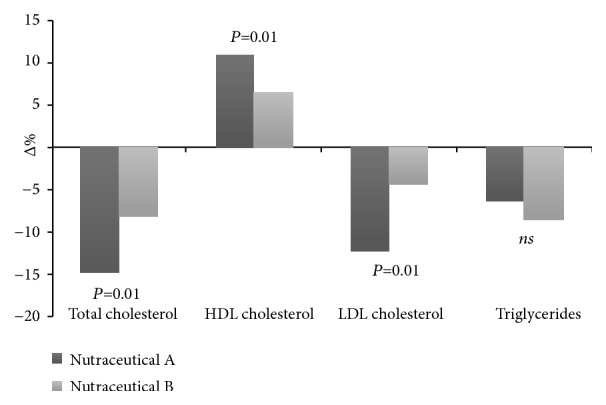
Percentage changes in lipid profile between baseline and follow-up.

For these measurements, we considered the percentage change found by both nutraceuticals associations versus the baseline values (Figure 2). Nutraceutical “A” reduced the total and LDL cholesterol more than nutraceutical “B”; similarly, participants in nutraceutical “A” group showed a higher improvement in HDL cholesterol level. On the contrary, no statistically significant modification was noted in the percentage changes of triglycerides between the two nutraceuticals mixtures.

Furthermore, according to ESC/EAS guideline [12], the rate of participants showing a redaction of LDL cholesterol below 115 mg/dl at the end of treatment periods has been calculated.

**Figure 3:**
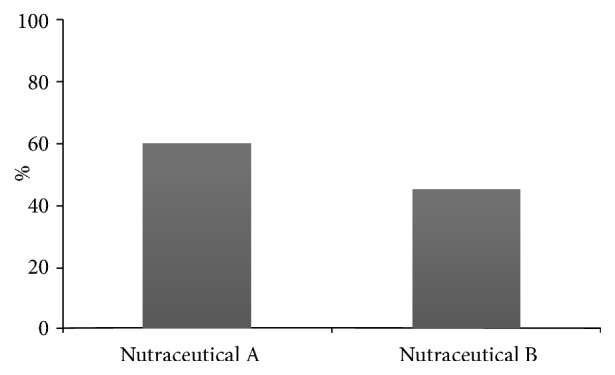
Percentage of subjects with LDL<130 mg/dl at the end of follow-up.

A greater number of subjects with a normal value of LDL cholesterol with nutraceutical “A” were observed as compared to nutraceutical “B” (59.6% versus 40.4 %, p=0.04) (Figure 3).

Finally, no statistically significant difference was observed for glucose, insulin, and HOMA index recorded throughout the study.

## 4. Discussion

This randomized study corroborates that treatment with specific nutraceutical combination (actually approved in Italy) is able to ameliorate the lipid profile among subjects with high levels of cholesterol not specifically needing statins. Moreover, it suggests that the nutraceutical containing 5 mg of monacolin K, 200 mg of the extract* Citrus bergamia*, 400 mg of Omega-3, and 10 mcg of trivalent chromium entailed a cholesterol lowering effect with a significant improvement of endothelial function. This last effect is very important, in particular considering that endothelial dysfunction is considered an early event of atherosclerosis that precedes structural atherosclerotic changes in the vascular wall [18] and several studies have demonstrated that impaired FMD predicts CV morbidity and mortality independently of traditional CV risk factors and the Framingham risk score [19, 20]. These findings have been recently confirmed also in young population, in absence of CV disease [21].

As recommended by the 2016 ESC/EAS guidelines for the management of dyslipidaemias, nutraceuticals containing purified red yeast rice may be considered in subjects with elevated plasma cholesterol levels who do not qualify for treatment with statins in view of their global CV risk [20]. The bioactive ingredient of red yeast rice is the monacolin K. Effects of monacolin K on lipid profile are correlated to a statin-like action with the inhibition of hydroxymethylglutaryl-coenzyme A (HMG-CoA) reductase. Diverse nutraceutical combinations have different concentrations of monacolin K, while a clinically relevant effect on cholesterol levels (up to a 20% reduction) is observed with preparations providing a daily dose of monacolin K between 2.5 to 10 mg [22–24]. The higher concentration of monacolin K in the mixture “A” may in part explain the better results obtained in the control of lipid profile and on the endothelial function. However, it is possible to hypothesize that these better results may be explained by the presence of other specific ingredients, too.

The extract of bergamot (*Citrus bergamia*) is rich in flavonoids that have antioxidant, anti-inflammatory, as well as lipid-lowering properties [25]. The hydroxy methyl-glutaryl-flavonones contained in the* Citrus bergamia*, such as hesperetin, naringenin brutieridine, and melitidine, have specific effects on lipid profile inhibiting, as the statin, the enzyme HMG-CoA reductase, and the activity of AcylCo-enzymeA Cholesterol-Acyl-Transferase (ACAT), then reducing the assembly of VLDL and LDL lipoproteins [26]. Furthermore, as regards the potential effect on the endothelium, the flavonoids contained in the* Citrus bergamia* limit the production of oxygen free radicals in the vessel wall, improve the endothelial production of nitric oxide, and also modulate the inflammatory response. In particular, by inhibiting the activation of NF-kB, they reduce the expression of the inflammatory cytokines, such as IL-6, IL-1b, TNF-alpha, and therefore have antiproliferative effects on the smooth muscle cells and contribute to the prevention of atherosclerosis [27].* Citrus bergamia* can therefore play an important role in preventing vascular damage due to proliferation of smooth muscle cells and dysfunction of endothelial cells. A study of an angioplasty model in rats showed that pretreatment with* Citrus bergamia* reduced neointima proliferation, free radical formation, and LDL-ox receptor expression [25]. Toth and colleagues [28] recently investigated the effects of* Citrus bergamia* flavonoids supplementation on cardio-metabolic risk in dyslipidemic subjects, demonstrating that this supplementation significantly reduced plasma lipids and improved the lipoprotein profile. Carotid intima-media thickness was also reduced significantly over a relatively short time frame of 6 months [28].

Trivalent chromium (Cr3+) is classified as an essential micronutrient for optimal carbohydrate and lipid metabolism. Although evidence relating Cr3+ deficiency and CV disease is incomplete, deficit has been associated with lower levels of HDL cholesterol. Sealls and colleagues [28] demonstrated that Cr3+ reversed hyperinsulinemia-induced cellular cholesterol accrual and associated defects in cholesterol transporter ABCA1 trafficking and apolipoprotein A1-mediated cholesterol efflux. These findings suggest a mechanism of Cr3+ action that fits with long-standing claims of its role in cholesterol homeostasis [29].

The lipid-lowering action of omega-3 polyunsaturated fatty has been clearly demonstrated in numerous clinical trials and meta-analyses [30, 31]. Nutraceuticals with omega-3 have a number of other beneficial effects on biomarker parameters that justify their use in subjects with metabolic syndrome [32]. In fact, they also stimulate the formation of prostaglandins, inhibit the activity of the enzyme to convert angiotensin, reduce the formation of angiotensin II and increase that of nitric oxide, and reduce the formation of homocysteine [33–35].

So, we can speculate that the final beneficial effect of nutraceutical “A” (*Citrus bergamia*, Omega-3 Crominex 3+, monacolin K; Riscol Plus, Errekappa Euroterapici) is the result of a series of synergies that are created between different nutrients. For these reasons, the components present in this nutraceutical should not be seen in an exclusively lipid profile optics, but rather for the anti-inflammatory, antioxidant, and vasodilation actions that they are able to perform.

Albeit dealing with a highly relevant result, our study presents several limitations that need to be discussed. First, this study should be considered a pilot trial showing a significant improvement of endothelial function and lipid profile; however, it is important to highlight that the small number of participants enrolled needs validation in a larger sample. The short follow-up limits the probability to observe positive results on insulin and glycemic levels, which needs a longer time of follow-up to assess the differences. Nevertheless, our preliminary data are promising and could be the base for a larger clinical trial.

Apart from these limitations, this study offers new evidence about the positive effect of functional food on lipid profile and endothelial function with a good compliance and the absence of significant side effects. Dietary recommendations should always take into account healthy food choices; however, innovative nutritional strategies to improve dyslipidaemias, such as nutraceutical combinations, should be promoted [35, 36]. In this respect it is important to highlight that the two formulations considered in the present study are very different in composition. This means that no specific conclusion regarding one or more compounds may be definitively reached and, as a consequence, a larger study specifically designed for this purpose will be needed.

## Figures and Tables

**Table 1:** Characteristics of study population according to gender *∗*.

**Characteristics**	**Total Sample** **(n=47)**	**Nutraceutical** **A** **(n=24)**	**Nutraceutical** **B** **(n=23)**	***p***
**Age** (years)	58.7± 8.7	58.0± 8.9	59.5± 8.5	0.55

**Gender** (male)	33 (70)	16 (67)	17 (74)	0.41
**Smoking**	7 (14)	4 (16)	3 (13)	0.45
**Healthy diet**	25 (53)	13 (54)	12 (52)	0.77
**Physically active**	19 (40)	10 (41)	9 (39)	0,65
**BMI **(Kg/m^2^)	27.3± 4.6	28.0± 5.1	26.5± 5.9	0.28
**Systolic blood pressure** (mmHg)	122,9± 14.0	125.8± 14.4	119.8± 13.2	0.14
**Diastolic blood pressure** (mmHg)	75.4± 9.9	76.4 ± 10.9	74.3± 8.9	0.47
**Total cholesterol** (mg/dl)	239.1± 26.3	234.9± 10.0	243.6± 36.1	0.29
**HDL cholesterol** (mg/dl)	47.0± 3.2	46.9± 2.0	47.2± 4.2	0.46
**LDL cholesterol** (mg/dl)	143.8± 25.6	141.3± 15.3	146.4± 33.3	0.49
**Triglycerides **(mg/dl)	105.4± 34.3	104.3± 40.3	106.5± 27.6	0.83
**Fasting plasma glucose** (mg/dl)	91.1± 6.4	90.2± 7.6	91.9± 5.0	0.38
**Insulin **(lU/ml)	8.8 ± 5.3	9.4 ± 5.8	8.1 ± 4.8	0.43

*∗*Data are given as number (percent) for gender, smoking, healthy diet, and physical activity; for all the other variables, means ± SD are reported.

Healthy diet: consumption of at least three portions of fruit and/or vegetables per day.

Physically active: physical exercise at least twice a week.

BMI: body mass index.

**Table 2:** Unadjusted and adjusted means of flow-mediated dilation (FMD) measures (dependentvariable) according to different treatments.

**Characteristics**	**Nutraceutical A** **(n=24)**	**Nutraceutical B** **(n=23)**	***p***
**Baseline**	5.19 ± 2.25	4.74 ± 1.67	0.14

**Follow-up **(unadjusted)	7.55 ± 3.38	5.75 ± 2.09	0.04
**Follow up **(adjusted) *∗*	7.48 ± 0.58	5.58 ± 0.59	0.05

*∗* ANCOVA: analysis adjusted for age, gender, and baseline value.

## Data Availability

The data used to support the findings of this study are available from the corresponding author upon request.
